# Strategic Design and Fabrication of Biomimetic 3D Scaffolds: Unique Architectures of Extracellular Matrices for Enhanced Adipogenesis and Soft Tissue Reconstruction

**DOI:** 10.1038/s41598-018-23966-3

**Published:** 2018-04-09

**Authors:** Afeesh Rajan Unnithan, Arathyram Ramachandra Kurup Sasikala, Shalom Sara Thomas, Amin Ghavami Nejad, Youn Soo Cha, Chan Hee Park, Cheol Sang Kim

**Affiliations:** 1 Division of Mechanical Design Engineering, Chonbuk National University, Jeonju, Republic of Korea; 2 Department of Bionanosystem Engineering Graduate School, Chonbuk National University, Jeonju, Republic of Korea; 3 Department of Food Science and Human Nutrition, Chonbuk National University, Jeonju, Republic of Korea; 4 Advanced Pharmaceutics and Drug Delivery lab, University of Toronto, Toronto, Canada

## Abstract

The higher rate of soft tissue impairment due to lumpectomy or other trauma greatly requires the restoration of the irreversibly lost subcutaneous adipose tissues. The nanofibers fabricated by conventional electrospinning provide only a superficial porous structure due to its sheet like 2D structure and thereby hinder the cell infiltration and differentiation throughout the scaffolds. Thus we developed a novel electrospun 3D membrane using the zwitterionic poly (carboxybetaine-co-methyl methacrylate) co-polymer (CMMA) through electrostatic repulsion based electrospinning for soft tissue engineering. The inherent charges in the CMMA will aid the nanofiber to directly transform into a semiconductor and thereby transfer the immense static electricity from the grounded collector and will impart greater fluffiness to the scaffolds. The results suggest that the fabricated 3D nanofiber (CMMA 3NF) scaffolds possess nanofibers with larger inter connected pores and less dense structure compared to the conventional 2D scaffolds. The CMMA 3NF exhibits significant cues of soft tissue engineering such as enhanced biocompatibility as well as the faster regeneration of cells. Moreover the fabricated 3D scaffolds greatly assist the cells to develop into its stereoscopic topographies with an enhanced adipogenic property.

## Introduction

Soft tissue engineering (STE) is a burgeoning field that introduces one of the most important challenges in the biomedical research related to the various adipose tissue pathologies and defects^1,2^. The higher rate of soft tissue impairment due to lumpectomy or other trauma greatly requires the restoration of the irreversibly lost subcutaneous adipose tissues. The curative efforts of soft tissue defects are very crucial because of its adverse impact on the patients’ emotional well-being due to the cosmetic defects rather than the impaired function^3^. Though the autologous tissues or biocompatible biopolymer based fillers are currently used for breast reconstruction surgeries associated with breast cancer therapies with a considerable rate of clinical success, they have their own side effects due to the volume loss and donor site morbidity over time^3,4^. By overcoming these difficulties, the soft tissue engineering has shown promising potential to aid the development of large volume of soft tissue augmentation in reconstructive and cosmetic plastic surgery^5–7^.

Current STE approaches introduce the application of a bioactive scaffold which may carry specific cells, growth factors and other bioactive materials to function as the first artificial matrix layer in the tissue defect area. The scaffolds play a very crucial role in supporting the invading cells to create the new extra cellular matrix (ECM) and reassure them to attach and proliferate to form the new functional tissue in the area^8–10^. The ultimate tissue engineering scaffold would be able to closely mimic the structure and the spatial topographies of natural ECM to support the cells to grow and differentiate to the respective tissue^11–13^. Development of scaffolds with biological and mechanical properties supportive to native adipose tissue is still a challenge for the tissue engineering researchers. Electrospun 3D scaffolds will be the best available choice for STE due to its unique surface properties such as high surface area to volume ratio, variability in pore size distribution and higher porosity etc.^14,15^.

Electrospun nanofiber scaffolds have gained much attention as a promising material for soft tissue engineering applications due to their structural similarity to mimic the architecture of the natural ECM. Moreover the nanofiber scaffolds can control the cell phenotype, initiate cell to ECM communications and can provide an inducing platform for cell adhesion, proliferation and differentiation^16,17^. Recent studies reported that the cells cultured on 2D cell adhesion platforms may differ in morphology and differentiation pattern compared to those cultured in a physiological 3D substrate^18^. Hence it is reasonable to fabricate scaffolds with 3D structures with particular morphological features and configurations according to the characteristics and functions of the tissues of interest^19^. Generally the electrospun nanofibers create a 2D scaffold with nanofibers arranged either random or aligned to the collector and most possibly will develop into flat shapes^20,21^. The function and the differentiation strategy of the cells growing on these flat scaffolds may not be similar that of the original native tissues.

Recently a few studies were reported on the electrostatic repulsion based fabrication of 3D electrospun scaffolds. In most of the reported studies electrospinning was done with integration of coarse fibers or electrospinning with porogens or the addition of conducting materials etc. to invoke the 3D structure^22,23^. These techniques could substantially increase the distance between the electrospun fibers and can lead to the comparable cell penetration but not exactly the same happen in most of the cases. Such fabrication technique may change the planar orientation of the electrospun fibers. In order to put up with these problems, we have developed a novel 3D electrospun scaffold based on the principle of electrostatic repulsion based electrospinning. The zwitterionic poly (carboxybetaine-co-methyl methacrylate) co-polymer termed as CMMA was synthesized and exploited for repulsion electrospinning to fabricate the 3D scaffolds (CMMA 3NF). The zwitterionic CMMA increase the surface charge of the nanofiber during the electrospinning process to induce the formation of electrospun 3D structures. The zwitterionic polymers were well known for tissue engineering applications due to their unique properties^6,24–27^. The CMMA 3NFs were further exploited to study the applicability for STE by evaluating the adipogenic property using 3T3L1 preadipocytes. 3T3L1 can promote angiogenesis by secreting VEGF (Vascular endothelial growth factor) and the vein formation of endothelial cells can be induced by the culture supernatant of 3T3L1 cells. In addition to that, the expression of Ang-1 (angiogenic factor-1) is higher when 3T3L1 preadipocytes are differentiated to adipocytes, indicating that the preadipocytes promote angiogenesis during adipogenic process. In this study we propose that the CMMA 3NF could facilitate the preadipocytes (3T3L1) to attach, proliferate and differentiate further confirming its applicability for STE.

## Results and Discussion

**Figure 1:**
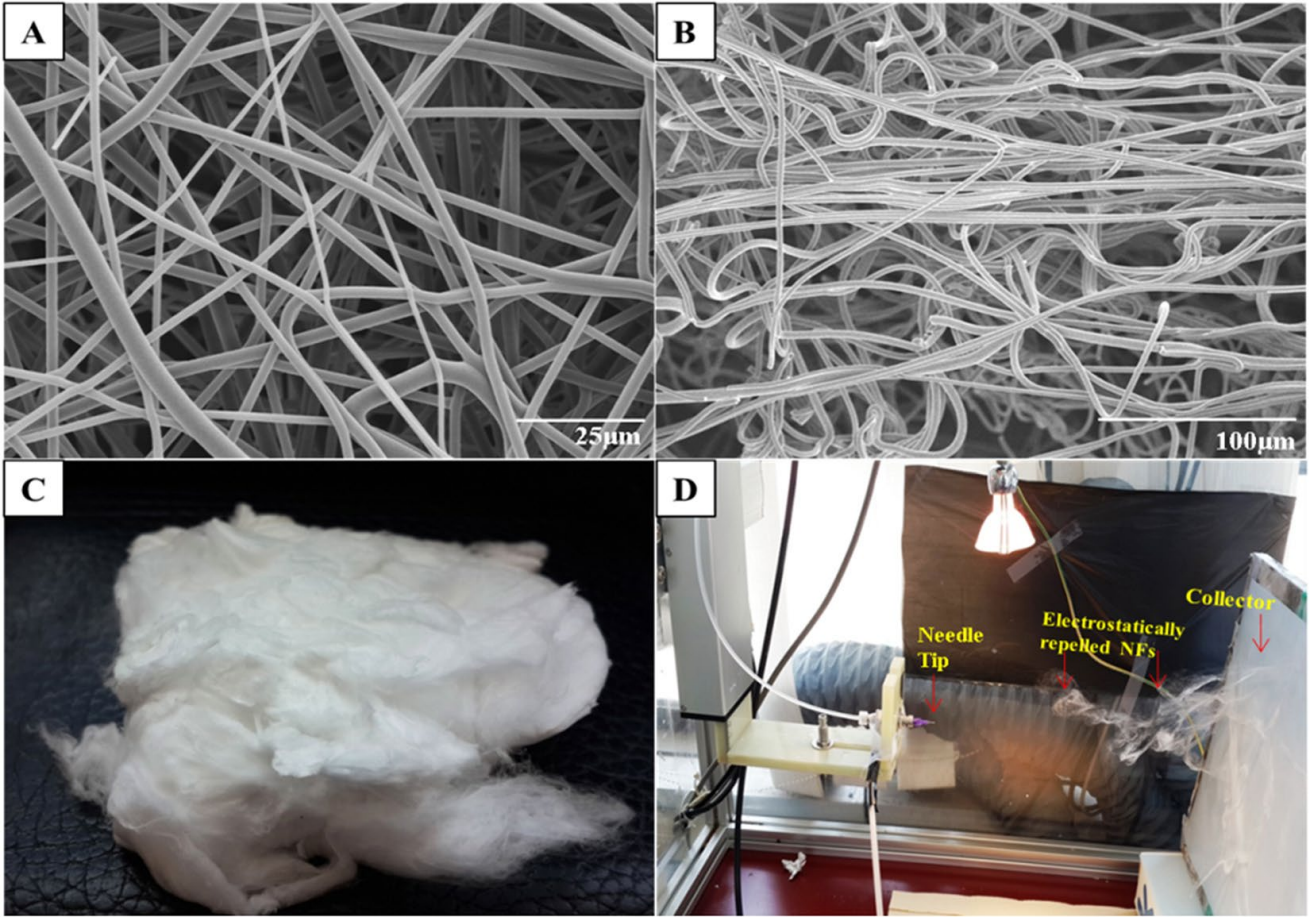
FESEM images of CMMA 3NF membrane (**A**) top view, (**B**) side view (**C**) digital photograph of CMMA 3NF scaffolds fabricated and (**D**) the digital photograph showing the formation of 3D nanofibrous scaffold during electrospinning.

The CMMA 3NF were successfully fabricated through the electrostatic repulsion electrospinning. The FESEM (Field Emission Scanning Electron Microscope) images (Fig. 1) showed the bead free randomly oriented nanofibers with uniform diameter (700–800 nm range, supporting information) and smooth surface. The FESEM images also showed that the CMMA 3NF scaffolds possess larger pores and less dense structure compared to the traditional electrospun scaffolds^28^. Moreover from the images it’s clear that in most of the areas the pore diameter exceeds more than 20μm. An optimal porosity is necessary for the cells to infiltrate and migrate deep into the interior of the scaffold which is crucial for the viability of the cells^29^. Poor infiltration of cells in to the 3D structure is a significant issue that seriously affects the success of the tissue engineering application of the electrospun scaffolds. So the development of CMMA 3NF scaffold can overcome the long existed problem of poor infiltration capability of cells in electrospun scaffolds. The porosity of the CMMA 3NF was calculated by mercury intrusion method and it was also confirmed the highly porous structure of CMMA 3NF scaffold.

**Figure 2:**
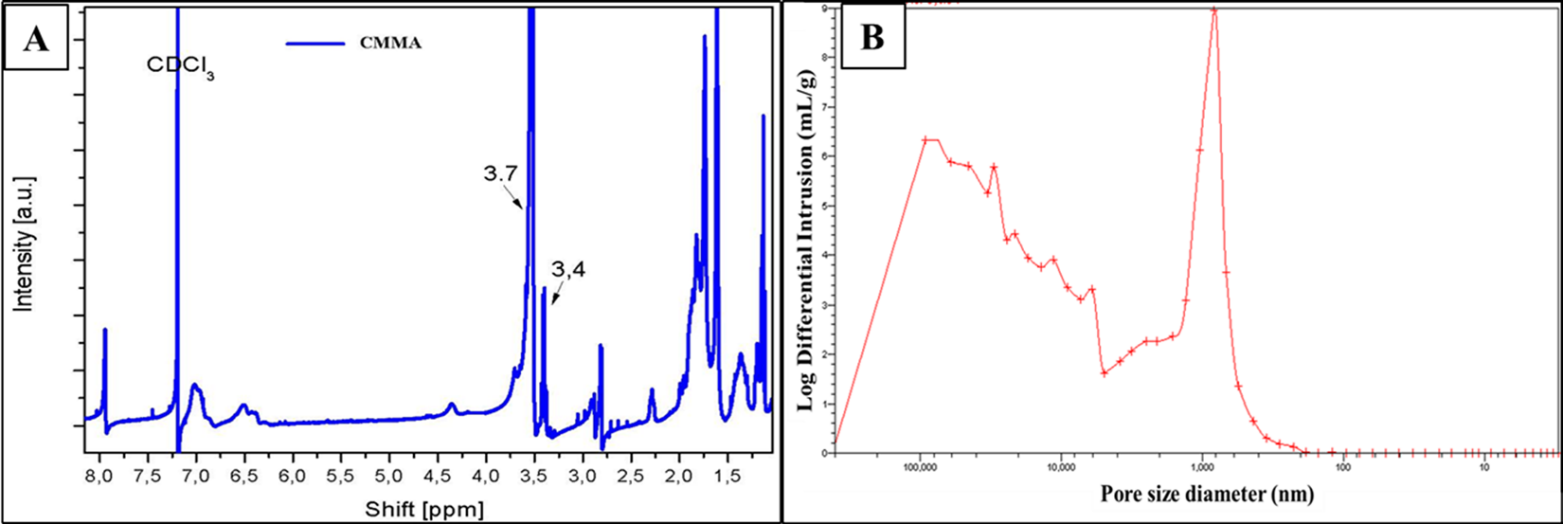
(**A**) ^1^HNMR data of copolymer, (**B**) graph of log differential intrusion (mercury) vs pore size of CMMA 3NFs.

Prior to mercury intrusion, the penetrometer was degassed to approximately 50 μm Hg to remove air from the system. The void space of the chamber was filled with mercury (initial pressure 0.043 psi). Logarithmically spaced data points were taken at pressures ranging from 0.1 to 60000.00 psi. For the purpose of data analysis, the surface tension of mercury and the intrinsic contact angle with the nanofiber were 485.00 dynes/cm and θ = 13° respectively. All the pore size distributions were corrected for deformation of the nanofiber mats under elevated pressure as described elsewhere^30^. As shown in Fig. 2B, the graph of log differential intrusion vs pore size of CMMA 3NF scaffold were plotted and analyzed. A mean pore diameter of 35.1205μm was observed in CMMA 3NF scaffold. The increased pore size in CMMA 3NF scaffold may be due to the electrostatic repulsion of the charged zwitterionic polymer during electrospinning, which eventually resulted in the formation of fluffy and porous CMMA 3NF scaffolds.

**Figure 3:**
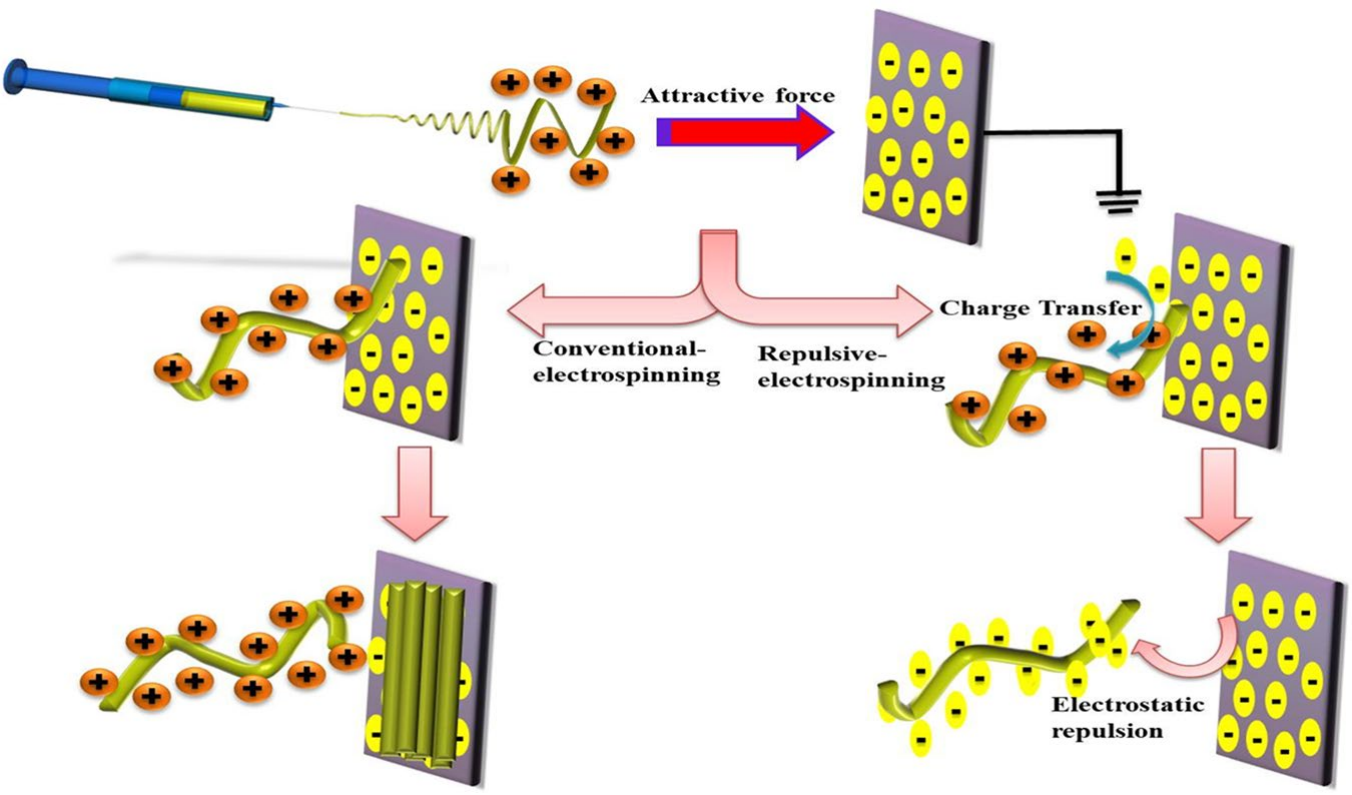
Proposed mechanism of formation of 3D nanofiber scaffolds compared to 2D nanofibers.

**Figure 4:**
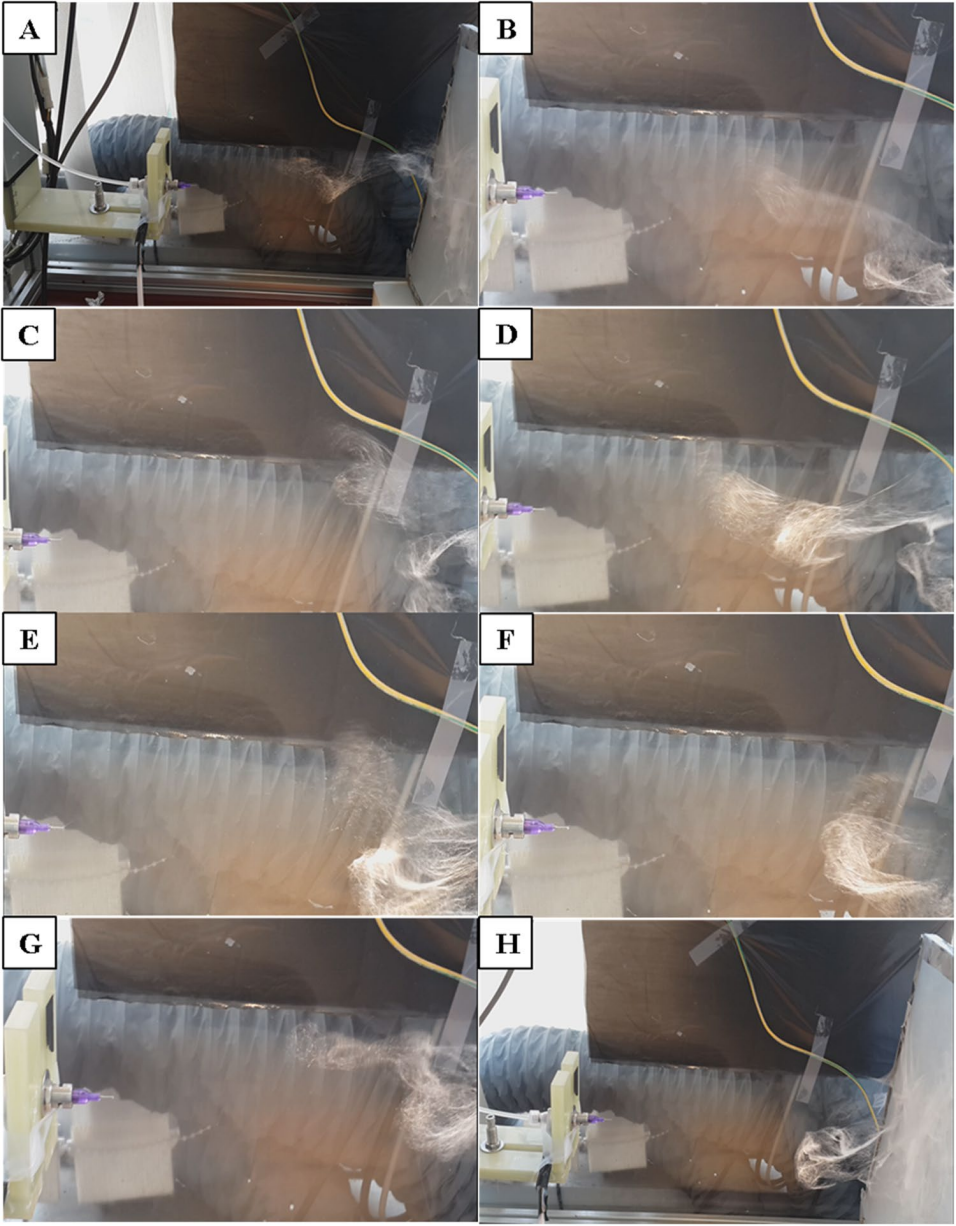
Digital photograph showing the different stages of electrostatic repulsion based electrospinning. The fibers touch the collector and repelled back and then deposited as fluffy nanofibrous scaffold is presented from (**A**–**H**) respectively.

It was reported that the low electrical conductivity or comparatively high surface electrical conductivity of the polymer can be the cause for the formation of 3D scaffolds during electrospinning^23,31^. During normal electrospinning, charge transfer from the fiber to collector at the time of their first contact is very rare due to the high surface resistivity of the fibers. The fibers with higher positive charges will be strongly attracted towards the grounded collector. It may be the reason that in the case of normal electrospun scaffolds the fibers will be oriented parallel to the collector in a 2D manner. But in case of electrospinning of 3D scaffolds as we did, the low resistivity of polymer fiber allows to transfer charges from the fiber to the collector as shown in scheme (Fig. 3). As the fiber touch the collector static surface charges will be transferred to the collector in a faster way and leaving less charge on the fibers and cause the decline in attraction between the fibers and collector. Moreover the fibers near to the collector can carry negative charges and will be repelled by the collector, although the tail end of the fiber were still attracted and moved towards the collector as shown if Fig. 4(A–H). In final the CMMA 3NF scaffolds will be obtained on the collector with fibers were arranged in several orientations and fluffy in manner. Compared to the convention 2D electrospinning, by introducing zwitterionic polymer the fiber was converted from insulator to semiconductor and the ability of transferring static electricity of the fiber has been tremendously increased and it may resulted in the formation of fluffy CMMA 3NF scaffolds.

The inhibition of bacterial infections is a crucial part to improve the success of any tissue engineering scaffold. The antibacterial properties of zwitterionic materials are already well known^32,33^. In the present study, the CMMA 3NFs showed comparatively good antibacterial property as shown in Supporting information Figure S3. The antibacterial activity of CMMA 3NFs may be due to the presence of small molecular quaternary ammonium compounds within the carboxy betaine^32^. They may bind to the outer membrane of bacterial cell and permeate into the bacteria and cause death. So the CMMA 3NFs will be expected to reduce the initial stage of infections after implantation. Moreover the CMMA3NF showed higher swelling rate and slow degradation rate during the study as shown in Supporting information Fig. 5.

**Figure 5:**
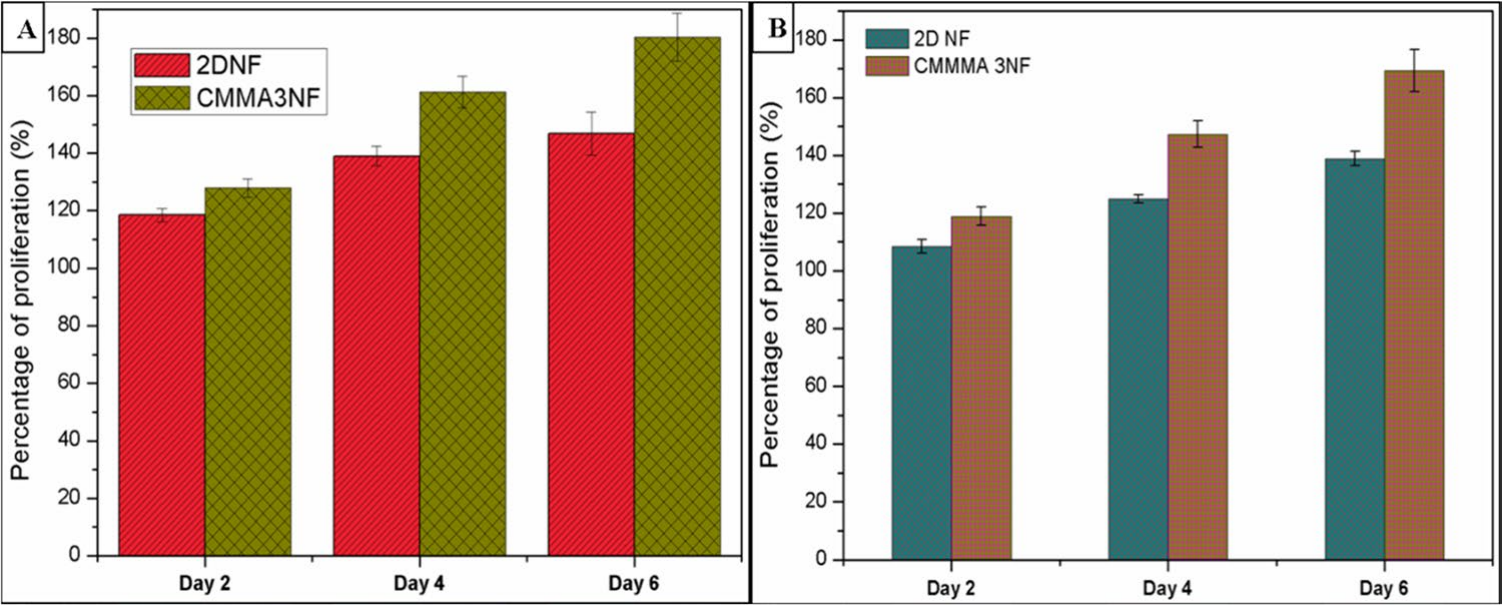
*In vitro* biocompatibility assay of 2D NFs and CMMA 3NFs on (**A**) MCF7 cell line and (**B**) 3T3L1 cell lines.

The *in vitro* biocompatibility studies showed that the CMMA 3NF scaffolds were well in supporting the growth of cells. In CMMA 3NF scaffolds the cells showed a remarkable proliferation rate as compared to 2D nanofiber mats. The MCF7 cell lines and 3T3L1 cells were able to penetrate, attach and proliferate in the 3D environment of CMMA 3NF scaffolds which may resulted in higher cell growth as seen in Fig. 5A and B.

**Figure 6:**
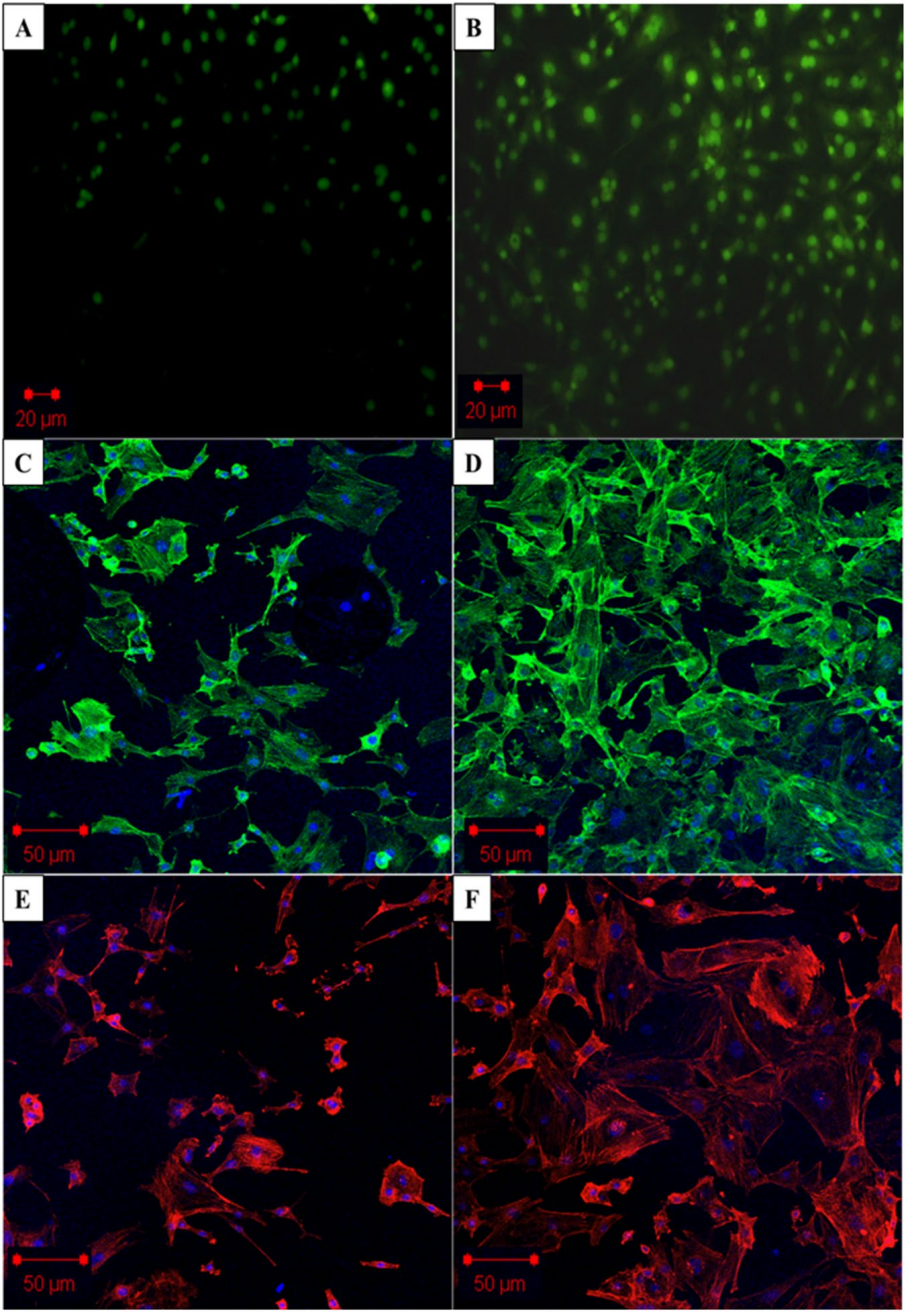
Confocal laser scanning microscopy (CLSM) showing the *in vitro* cell proliferation on CMMA 3NF. (**A**) Live/dead assay on day 2. (**B**) Live/dead assay on day 4, (**C**) Actin Green staining (**C**,**D**) and Rhodamine (**B**) staining (**E**,**F**) showed the cell viability on CMMA 3NF on day 2 and day 4 respectively.

Live/Dead assay also confirmed the higher number of live cells after 4 days, a strong indicator of material property to support and maintain cell growth, Fig. 6A. The confocal image results were also consistent with the biocompatibility results obtained. The Phalloidin green (Fig. 6C,D) and Rhodamine B (Fig. 6E,F) staining on the cells grown on CMMA 3NF scaffolds showed a very significant increase in cell number after 4 days. The unique structure of CMMA 3NF scaffolds with higher number of interconnected large pores could enhance the biocompatibility and the faster regeneration of cells, which is advantageous in tissue engineering and regenerative medicine^34,35^.

**Figure 7:**
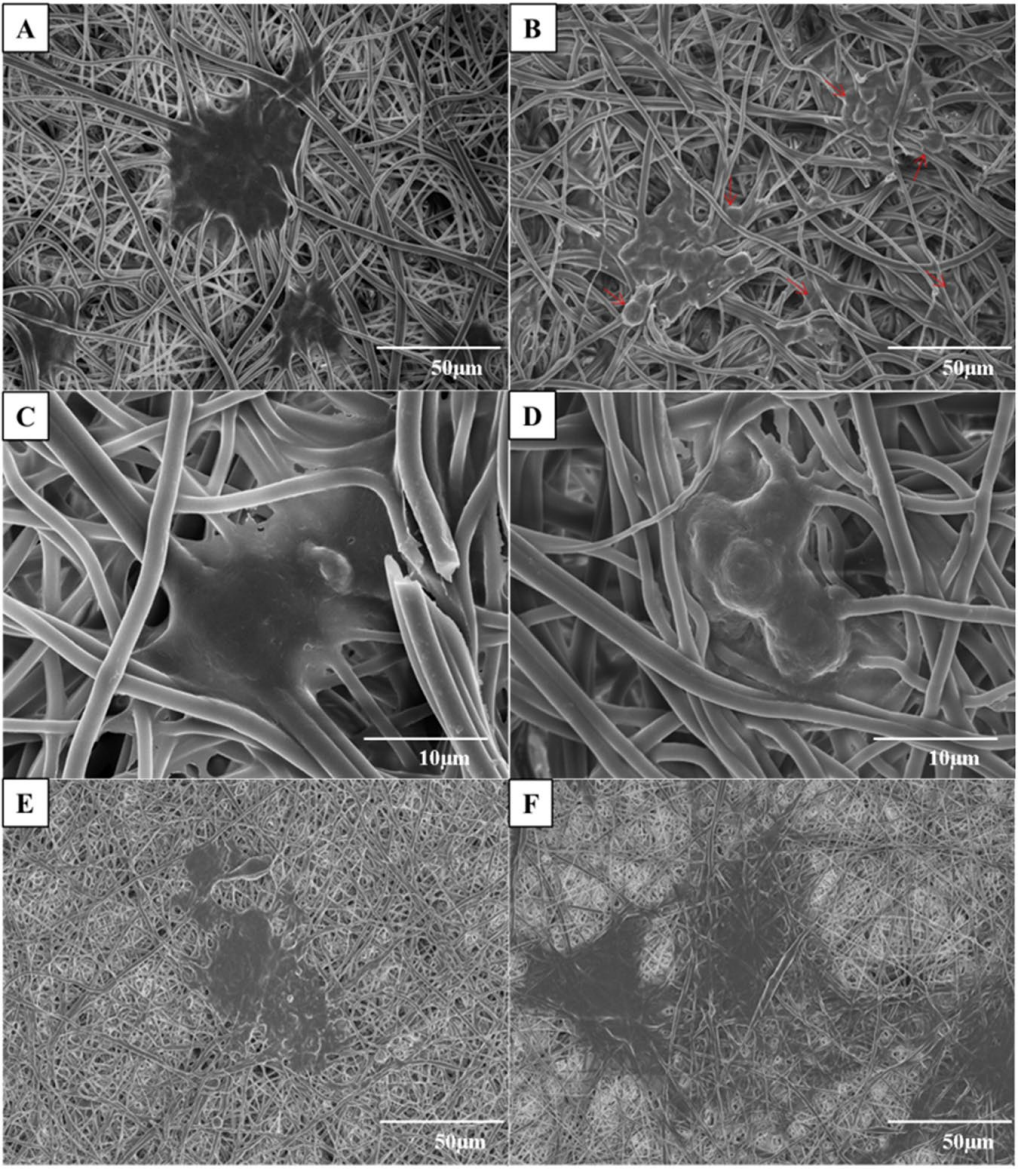
FESEM images showing the cell attachment and proliferation on CMMA 3NF on day 2 and day 4 (**A**,**B**), higher magnification image showing formation of cellular spheroid structure on day 2 and day 4 (**C**,**D**), Flattened cell morphology in 2D NF on day 2 and day 4 (**E**,**F**).

The attachment and proliferation of 3T3L1 cells in the CMMA 3NF scaffolds were observed through FESEM imaging for better understanding the cell behaviour on 3D scaffolds. Interestingly the cells grown on CMMA 3NF scaffolds attained more spheroids like morphology compared to the flattened cells formed on conventional 2D scaffolds. The morphological difference between the cells cultured on 2D and 3D scaffolds is a strong indication of CMMA 3NF scaffolds ability to support the cell growth in a 3D manner. The cells are become more spheroid as the day of culturing increases. As seen in Fig. 7D, the cells become more spheroid structure in day 4 as compared to day 2 and the cells were penetrated into the CMMA 3NF scaffold and formed the cellular spheroid structure as indicated with arrows in Fig. 7B.

**Figure 8:**
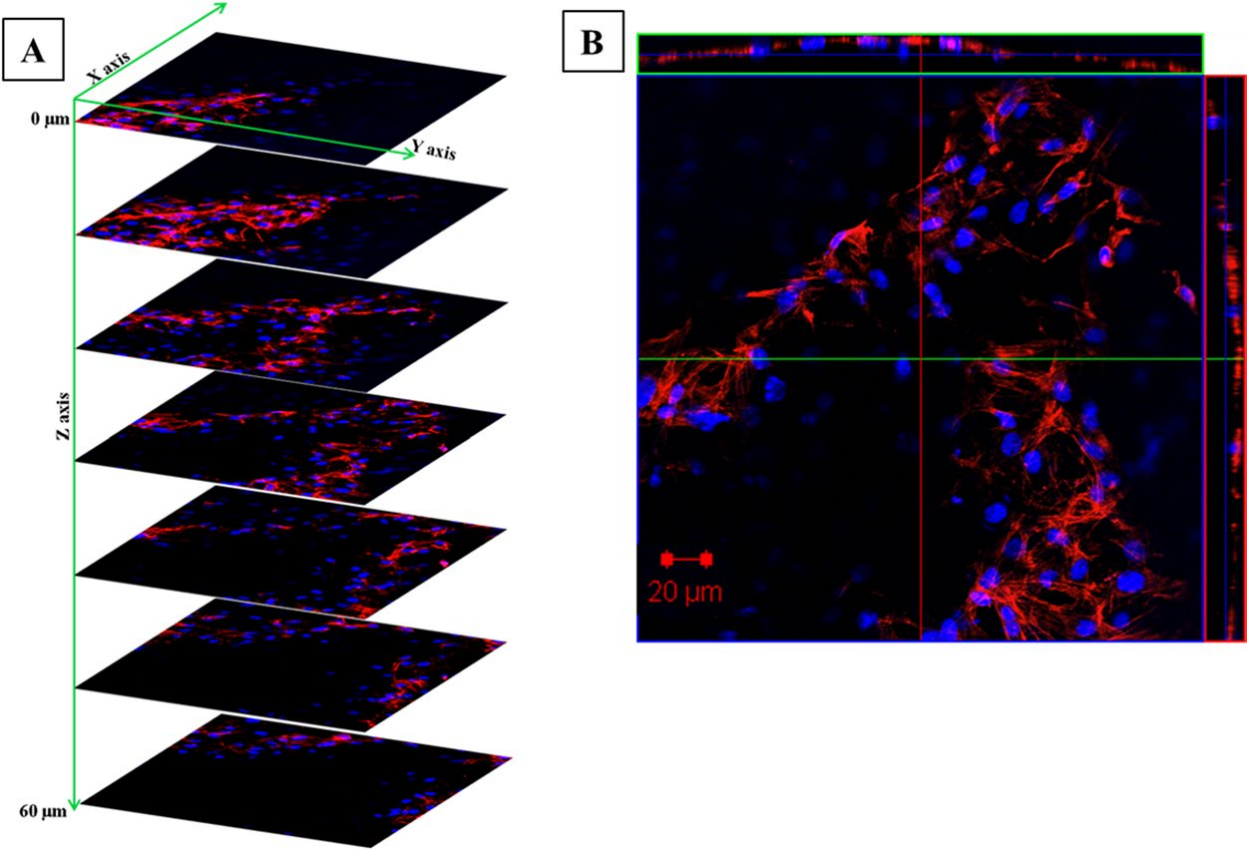
The confocal Z stacking images showing the cell infiltration in CMMA 3NF stained with Rhodamine (**B**) and DAPI.

But in 2D electrospun scaffold, the dense packing of nanofibers restricts the penetration of cells in the vertical manner and resulted in growing planar morphologies which are entirely different from its normal topography. As seen in Fig. 7E,F, in the 2D mat the cells become more flat structure by day 4 and spreads in a 2D manner. The confocal Z stacking imaging also confirmed the cell penetration into the CMMA 3NF scaffold. As seen in Fig. 8, the cells were penetrated more than 60 μm depth into the scaffold facilitating the cells to maintain its topological orientation. In tissue engineering aspects, the porosity and pore interconnectivity can directly control the cell infiltration. Thus the CMMA 3NF with higher interconnected pores that closely mimic the natural ECM can promote cell adhesion and proliferation and differentiation^36^. The obtained data suggests the promising application of CMMA 3NF for soft tissue engineering application.

**Figure 9:**
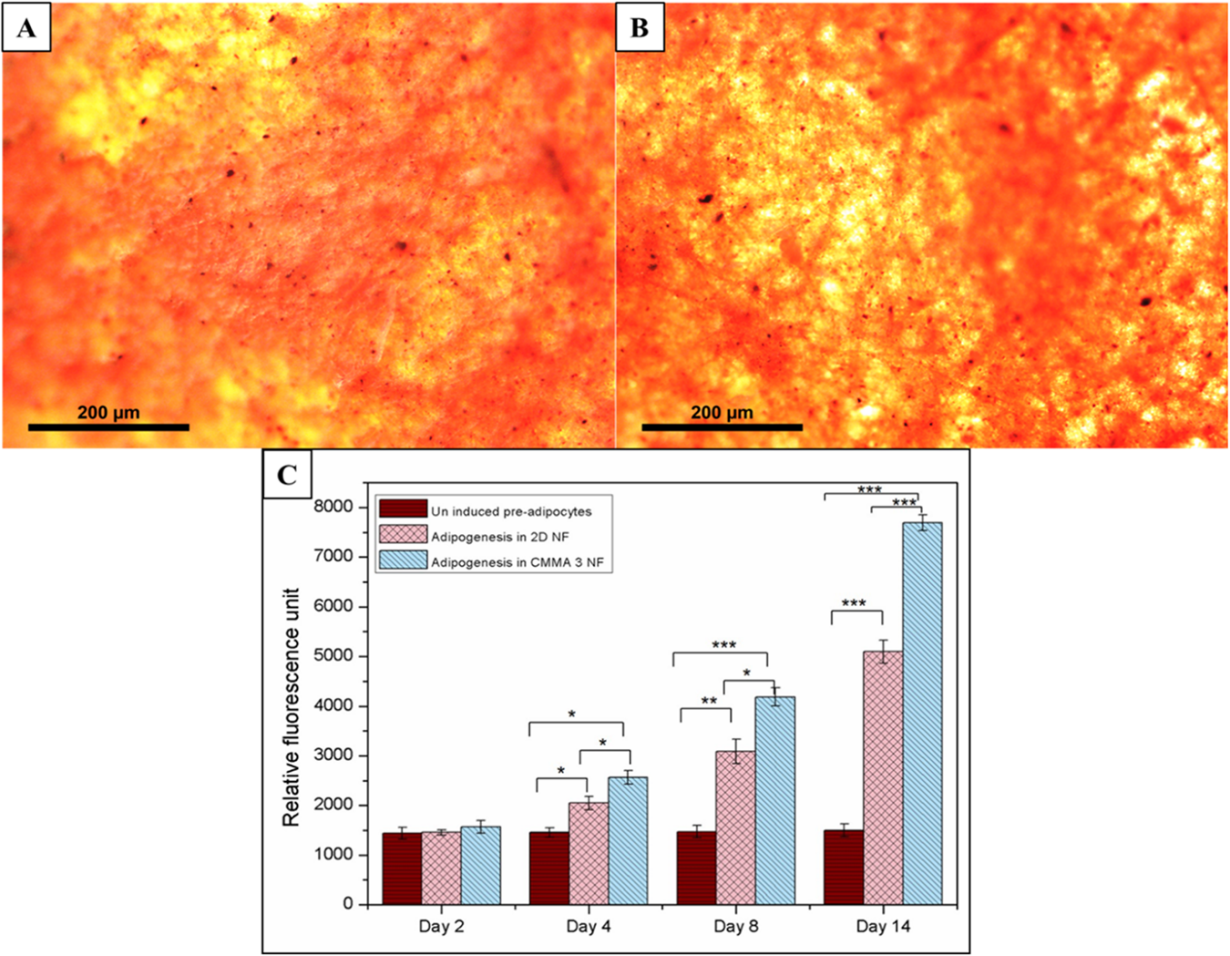
Oil Red O staining showing the lipid accumulation on CMMA 3NF (**A**) top view, (**B**) side view and (**C**) AdipoRed^TM^ assay showing the *in vitro* adipogenesis in 2D NF and CMMA 3NFs (Asterisks indicate a significance using ANOVA test [*P < 0.05; **P < 0.01; ***P < 0.001]).

The *in vitro* adipogenesis of 3T3L1 cells in CMMA 3NF was evaluated qualitatively by the Oil Red O staining and quantitatively through the fluorescence based AdipoRed^TM^ assay as shown in Fig. 9. The Oil Red O staining showed a very clear evidence for the ability of CMMA 3NF to support adipogenesis. The Oil Red O staining for the lipid accumulation is considered to be the golden standard for detecting adipogenesis *in vitro*. The staining showed that the CMMA 3NF can significantly support the soft tissue reconstruction by indicating more lipid accumulation in cells compared to the uninduced samples (Fig. 9A,B). The images of the cells stained with the Oil Red O on CMMA 3NF at different views were shown in Fig. 9A,B. The thick layer of fat tissue with red color is clearly visible in the top layer whereas the red dots of lipid accumulation are evident in the side view confirming the adipogenesis in CMMA 3NFs. As a quantitative measurement AdipoRed^TM^ assay is also performed to evaluate the adipogenesis as given in Fig. 9C. The results showed that the CMMA 3NFs possess higher intracellular lipid content compared to the 2D nanofiber mats. This was mainly due to the ability of preadipocytes to attach, penetrate and differentiate in the CMMA 3NFs more effectively compared to the dense 2D mats. Thus the AdipoRed^TM^ assay also confirmed the superior ability of CMMA 3NFs to support preadipocytes differentiation.

**Figure 10:**
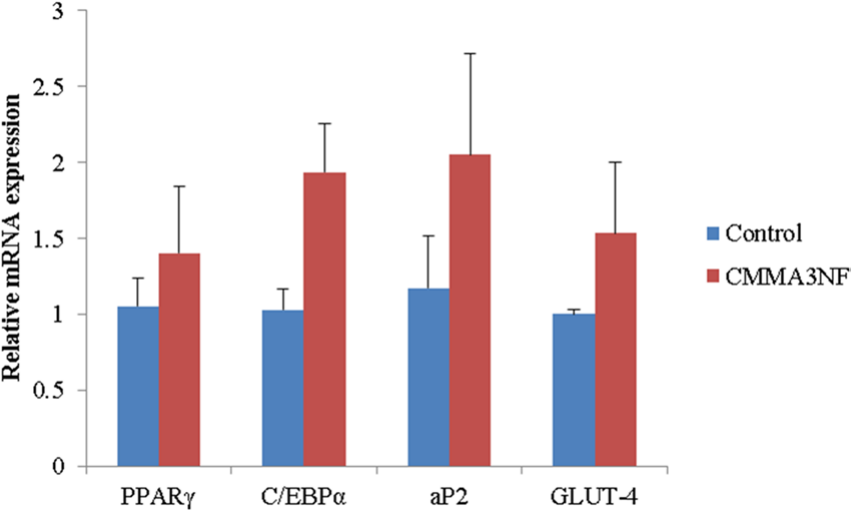
PCR gene expression study confirming the adipogenesis in CMMA3NF.

**Figure 11:**
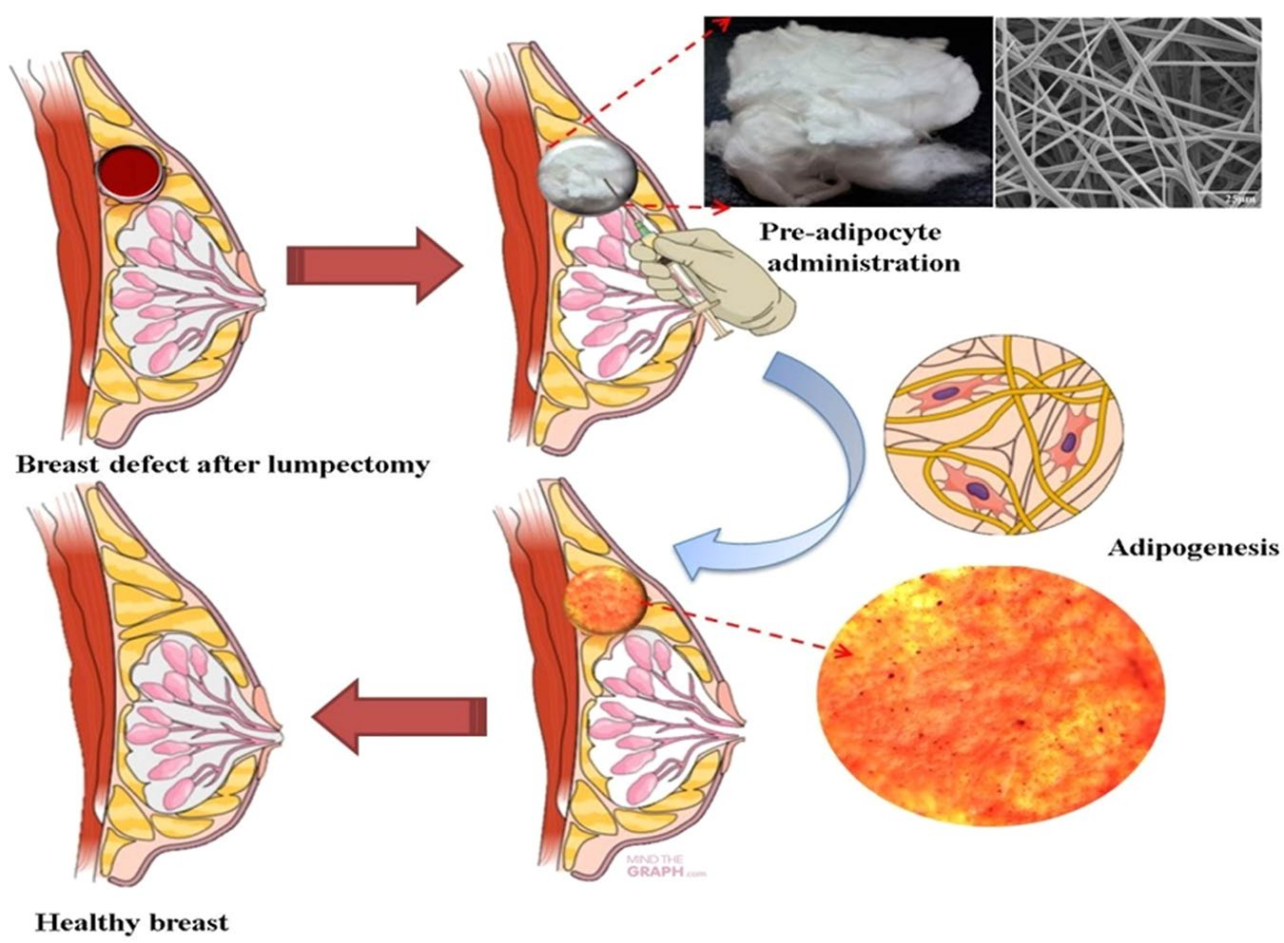
The proposed application of the 3D scaffold.

The PPARγ and C/EBPα are the transcription factors that regulate adipogenesis, and their expression determines adipose differentiation^37^. These transcription factors activates the genes involved in adipogenesis, such as aP2 and GLUT-4, which plays a major role in the late stage of adipocyte differentiation and regulates fatty acid storage leading to the formation of lipid droplets in the cells. The expression of aP2 and GLUT-4 are indicators late stage of differentiation^37,38^. In this study, we checked the expression of these genes involved in adipogenesis to confirm the differentiation of adipocytes. As shown in Fig. 10, the mRNA levels of the adipogenic genes in the cells grown with the sample were similar to those of the cells grown without the sample and these results were in agreement with the oil red O staining of cells in the sample, confirming that the cells were differentiated. The proposed application of the 3D scaffold is illustrated in Fig. 11.

## Conclusions

So here we successfully developed 3D nanofiber scaffolds with higher porosity and interconnected pores through the electrostatic repulsion mediated electrospinning. The fabricated scaffolds were able to mimic the 3D structure of the natural ECM and hence the cells were able to penetrate into the CMMA 3NF scaffold and formed the cellular spheroid structure. Moreover the CMMA 3NF scaffolds showed excellent adipogenic property, which is confirmed by the preadipocytes differentiation. The qualitatively evaluation by the Oil Red O staining and the quantitatively measurement by AdipoRed^TM^ assay and PCR analysis also showed the potential of CMMA 3NF to be an ideal material for soft tissue engineering application.

## Methods

### Synthesis and characterization of CMMA co-polymer

The monomer carboxybetaine was synthesized as reported previously^32^. Shortly N-(-Dimethylaminopropyl) acrylamide (50 mmol), ethyl 6-bromohexanoate (55 mmol) and acetonitrile (25 ml) were stirred at 45 °C under nitrogen blanket for five days. The product was precipitated using a rotary evaporator and washed with anhydrous ether. The monomers obtained (100 mg/ml) were dissolved in 1 M NaOH solution for 24 hr to hydrolysis of the ester groups and then neutralized with dilute HCL solution and vacuum dried.

^1^H-NMR (400 MHz, D2O): 2.00 (m, 4H, C-CH_2_-C), 2.47 (t, 2H, CH_2_-C=O), 3.1 (s, 6H, N^+^(CH_3_) _2_, 3.3–3.4 (6H, CH-N and CH_2_-N^+^ -CH2), 5.75 (m, 1H, CH=C-CON-trans), 6.19 (m, 1H, CH=C-CON-cis), 6.26 (m, 1H, =CH-CON-).

The two monomers methyl methacrylate (MMA, 47 mmol) and carboxybetaine monomer (4.7 mmol) were added to 30 ml of Dimethylformamide (DMF) solution. Under nitrogen atmosphere azobisisobutyronitrile was added to the above solution and heated at 70 °C and kept for overnight stirring. The obtained solution was carefully added dropwise to the diethyl ether (400 ml) under continuous stirring to precipitate the copolymer obtained. The obtained precipitate was vacuum dried at room temperature and it resulted in a yellowish polymer with a yield of 68%. The structure of the copolymer was confirmed by ^1^H-NMR (Fig. 2). In the obtained copolymer, the carboxybetaine to MMA molar ratio was estimated to be 9.8% by taking the integral of a peak at 3.4 ppm corresponds to the six protons present in the two methyl groups attached to the quaternary amine of carboxybetaine pendant group and at 3.7 ppm peak belongs to the three protons present in the terminal methyl group of the MMA pendant group. The materials were also tested chromatographically, but because of the presence of charge groups and poor solubility of the materials, it was not possible to provide exact values, even with the addition of salts such as LiCl. However, since the polymer solution is electrospinable it can be said the molar masses are high enough for the electrospinning process.

### Fabrication of electrospun 3D scaffold

The electrospun 3D dimensional nanofibrous scaffolds were developed through the custom made electrospinning setup and procedures reported already^21^. In brief, 20 wt% CMMA copolymer solution was prepared in DMSO and the solutions were taken in plastic syringe tube and were introduced to the metal capillary nozzle with diameter of 0.21 mm (21G), which is attached to a 1D robot system that moves laterally and is controlled by the Lab View 9.0 program (National Instruments). A continuous feed rate of 1 ml/hr is maintained during th experiment with a continuous voltage of 18KV and tip to collector distance was maintained at 15 cm. The control 2D nanofibers were fabricated using same electrospinning technique as described previously with 10 wt% polyurethane (PU) solution. Thus obtained PU nanofibers were vacuum dried and used as control 2D mat for further studies.

### Characterization studies

The surface morphology of the prepared CMMA 3NF scaffolds was observed by field emission scanning electron microscopy (FESEM; Zeiss Supra 40VP). The samples were attached on a carbon tape and sputter coated with platinum to deliver conductivity and decrease electrostatic surface charging and imaging was done. The pore size of the 3D nanofiber scaffolds were measured using mercury porosimetry (AutoPore IV series, Model no. 9500, Micromeritics, USA).

### Anti-bacterial studies of CMMA 3NFs

Escherichia coli (KACC 14818) and Staphylococcus aureus (KACC 10768) were purchased from Korea type culture collection. The antibacterial test of CMMA 3NFs and 2D NFs were done using the disc diffusion method^39^. Both materials were cut into small circular shapes of diameter 5 mm. These discs were placed on the agar medium and inhibition was studied for 240 min at 37 °C. The diameter of the zone of inhibition was measured.

### Swelling ratio and degradation evaluation of CMMA3NF

The CMMA3NF scaffolds with equal weights were immersed in PBS and kept at 37 °C and at predetermined time points the scaffolds were taken and swelling ratio was calculated as per the equation,

1 $${\rm{Swelling}}\,{\rm{Ratio}}=\frac{{\rm{Ws}}-{\rm{Wd}}}{{\rm{Wd}}}\,\times \,100$$

Ws and Wd denotes the wet and dry weights of the scaffolds respectively.

Similarly for degradation evaluation, the scaffolds were taken at regular time intervals and dried and the weight loss ratio were calculated as,

2 $${\rm{Weight}}\,{\rm{loss}}\,{\rm{ratio}}=\frac{{\rm{Wi}}-{\rm{Wd}}}{{\rm{Wi}}}$$

Wi and Wd denotes the initial and final weight of the CMMA3NF after incubation.

### Biocompatibility studies

The fabricated CMMA 3NF scaffolds were seeded with 3T3L1 and MCF7 (3 × 10^4^) in a 24 well plate and cultured in DMEM medium in 5% CO_2_ incubator at 37 °C in a humidified environment. The scaffolds were cut into 1.5 cm × 1.5 cm size to fit the cell culture plates and were taken in triplicate and the cell proliferation was analyzed in the alternative days up to 5 days using Dojindo’s cell counting kit-8 (CCK) assay. During 2^nd^, 4^th^ and 6^th^ day, CCK reagent was added and incubated for 4hrs and absorbances were measured using the microplate reader. Live/Dead cytotoxicity assay (Molecular probes, USA) was done to qualitatively analyze the cytocompatibility of the fabricated 3D scaffolds. The 3T3L1 preadipocytes were grown on the CMMA 3NFs for 6 days and were stained with 200 μl of calcein AM and ethidium homodimer (0.5 μM) for 30 minutes at room temperature. The CMMA 3NFs containing the cells were viewed directly under a fluorescent microscope with a standard fluorescein band pass filter for calcein and a Texas Red® dye filter for ethidium homodimer. The extent of 3T3L1 attachment and proliferation density (day 6) on the CMMA 3NFs were analyzed using the cytoskeletal Actin Green staining (Thermo Fisher Scientific, USA) and Rhodamine B (Molecular probes, USA) the cell nuclei were stained with DAPI (4,6-diamidino-2-phenylindole, Invitrogen, USA, 405 nm, blue).

### *In vitro* soft tissue engineering studies on CMMA 3NFs

The 3T3L1 preadipocytes were initially maintained in Dulbecco’s modified Eagle’s Medium (DMEM) supplemented with 10% fetal bovine serum (FBS), 100 µg/mL penicillin and 100 µg/mL streptomycin in a humidified atmosphere of 95% air/ 5% CO_2_ at 37 °C. The respective nanofiber samples were ultraviolet (UV) sterilized, rinsed in phosphate buffer saline (PBS) and soaked in cell culture media overnight prior to cell seeding. The cells were seeded to nanofiber samples with a cell density of 3 × 10^4^ cells/well and incubated at conditions suitable for cell growth. After 48 hours the medium was changed to differentiation medium supplemented with 10% FBS, 100 µg/mL penicillin, 100 µg/mL streptomycin, 1 µM dexathamethasone, 0.5 mM Methylisobutylxanthine (IBMX) and 10 µg/ml insulin (Sigma Aldrich, USA). After 48 hours the differentiation medium was replaced with Adipocyte maintenance medium supplemented with 10% FBS, 100 µg/mL penicillin, 100 µg/mL streptomycin and 1 µg/ml insulin and cultured until day 14.

### AdipoRed^TM^ Assay

The AdipoRed assay was conducted as per the manufactures instructions (Lonza, Walkersville, MD). The cells were collected from CMMA 3NF samples at indicated time points. The cells were suspended in 0.2 ml PBS in a 96 well plate along with 5 µl AdipoRed assay reagent and incubated for 15 minutes. After that the absorbance was measured at an excitation of 485 nm and the results were represented in relative fluorescence units (RFUs).

### Oil Red O staining

The CMMA 3NF containing the cells were fixed using 4% paraformaldehyde at predetermined time points and stained with Oil Red O (Sigma Aldrich, Korea) as per the protocol and then imaged under optical microscope.

### PCR analysis

3T3-L1 cells grown in 6- well plates with and without the CMMA3NF were differentiated after two days post confluency using the induction medium. After the differentiation period, the mRNA was extracted using Trizol reagent (Invitrogen Life Technologies, Carlsbad, CA, USA). After checking purity and concentration using Biodrop Duo instrument (Biochrom, Holliston,MA,USA), the mRNA was reverse- transcribed to cDNA using high-capacity cDNA reverse transcription kit (Applied Biosystems, Foster City, CA, USA). Then the expression levels of mRNA were quantified using quantitative real-time PCR using SYBER Green PCR Master Mix (Applied Biosystems, Warrington, UK) and a 7500 Real-Time PCR system (Applied Biosystems, Foster City, CA, USA) following the manufacturer’s protocol. Relative quantification of gene expression was calculated relative to GAPDH, the primers used are given as shown in Table 1.

**Table 1:** The primers used are given below.

Gene		Primers
PPARγ	Forward	5′GTGCCAGTTTCGATCCGTAGA-3′
	Reverse	5′-GGCCAGCA TCGTGTAGATGA-3′
C/EBPα	Forward	5′GTGTGCACGTCTATGCTAAACCA-3′
	Reverse	5′GCCGTTAGTGAAGAGTCTCAGTTT-3′
aP2	Forward	5′ – CCGATCCACTCCTTACCTCA-3′
	Reverse	5′-GCCACCGTGACCTTGTACTT-3′
GLUT-4	Forward	5′-GATTCTGCTGCCCTTCTGTC-3′
	Reverse	5′-ATTGGACGCTCTCTCTCCAA-3′
GAPDH	Forward	5′-TGCCACTCAGAAGACTGTGC-3′
	Reverse	5′-TTCAGCTCTGGGATGACCTT-3′

### Statistical Analysis

All the experiments were conducted in triplicate unless otherwise indicated and the results were indicated as mean +/− standard deviation. Analysis of variance (ANOVA) was used for statistical analysis and a p-value of <0.05 was considered statistically significant.

## Electronic supplementary material

Supplementary information
