# Supplementary material for: Strategic Design and Fabrication of Biomimetic 3D Scaffolds: Unique Architectures of Extracellular Matrices for Enhanced Adipogenesis and Soft Tissue Reconstruction
Source: Sci Rep. 2018 Apr 9;8:5696. doi: 10.1038/s41598-018-23966-3 (PMC5890269; doi:10.1038/s41598-018-23966-3)
Supplement: Supplementary file 1 — Supplementary information [file 41598_2018_23966_MOESM1_ESM.doc]

**Supporting Information**

**Strategic Design and Fabrication of Biomimetic 3D Scaffolds: Unique Architectures of Extracellular Matrices for Enhanced Adipogenesis and Soft Tissue Reconstruction**

**Afeesh Rajan Unnithan^1, 2#*^, Arathyram Ramachandra Kurup Sasikala^1, 2#^, Shalom Sara Thomas^3^, Amin Ghavami Nejad^4^, Youn Soo Cha^3^, Chan Hee Park^2*^, Cheol Sang Kim ^1, 2*^**

*^1^ Division of Mechanical Design Engineering, Chonbuk National University, Jeonju, Republic of Korea*

*^2^ Department of Bionanosystem Engineering Graduate School, Chonbuk National University, Jeonju, Republic of Korea*

*^3^Dept. of Food Science and Human Nutrition, Chonbuk National University, Jeonju, Republic of Korea*

*^4^Advanced Pharmaceutics and Drug Delivery lab, University of Toronto, Canada*

(# both authors contributed equally to the work)

***Corresponding authors**

Cheol Sang Kim ([chskim@jbnu.ac.kr](mailto:chskim@jbnu.ac.kr))

Chan Hee Park ([biochan@jbnu.ac.kr](mailto:biochan@jbnu.ac.kr))

Afeesh Rajan Unnithan ([afeesh@jbnu.ac.kr](mailto:afeesh@jbnu.ac.kr))


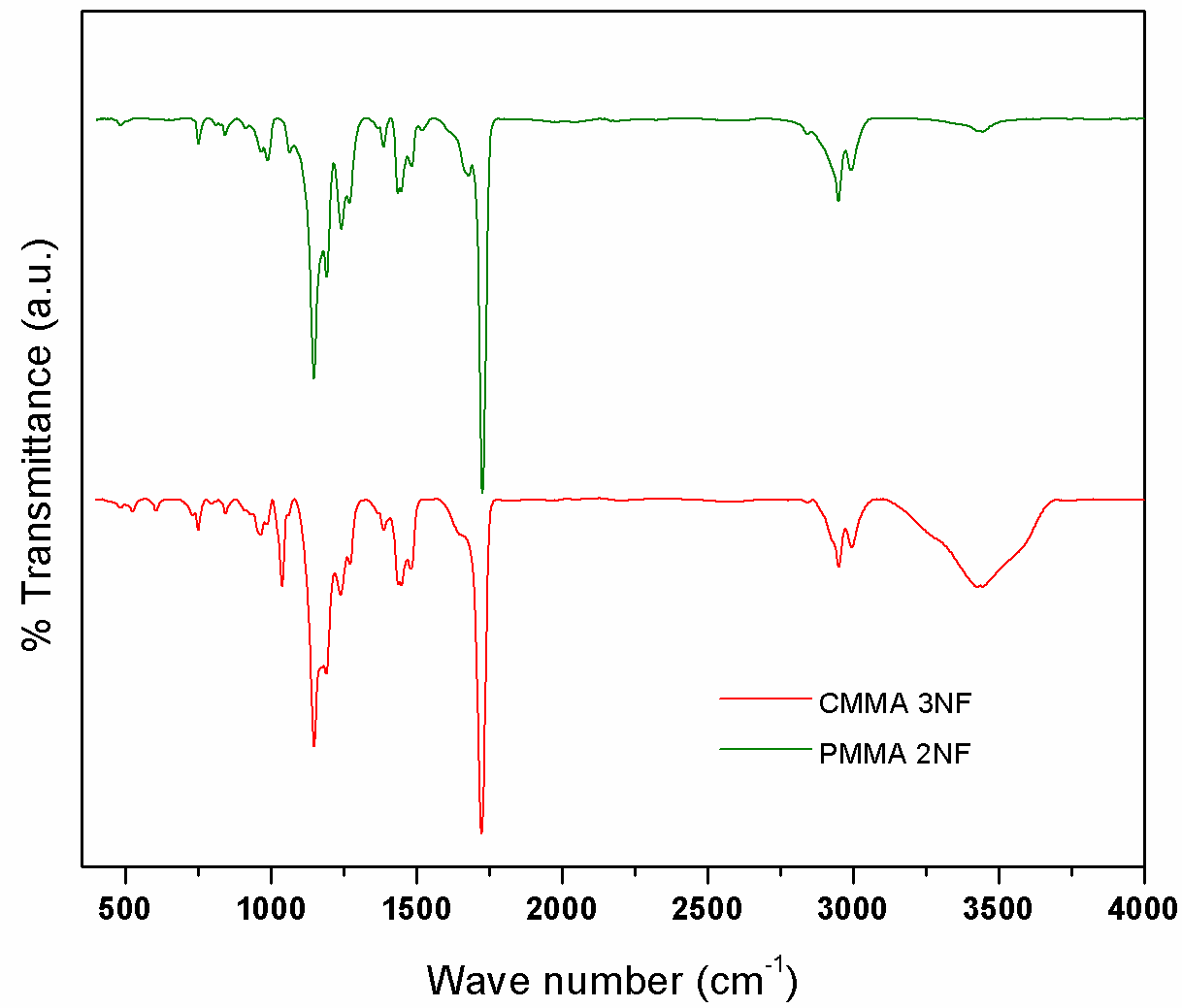


**Figure1.** FTIR data of CMMA3NF and PMMA 2NF mats showing the corresponding peaks


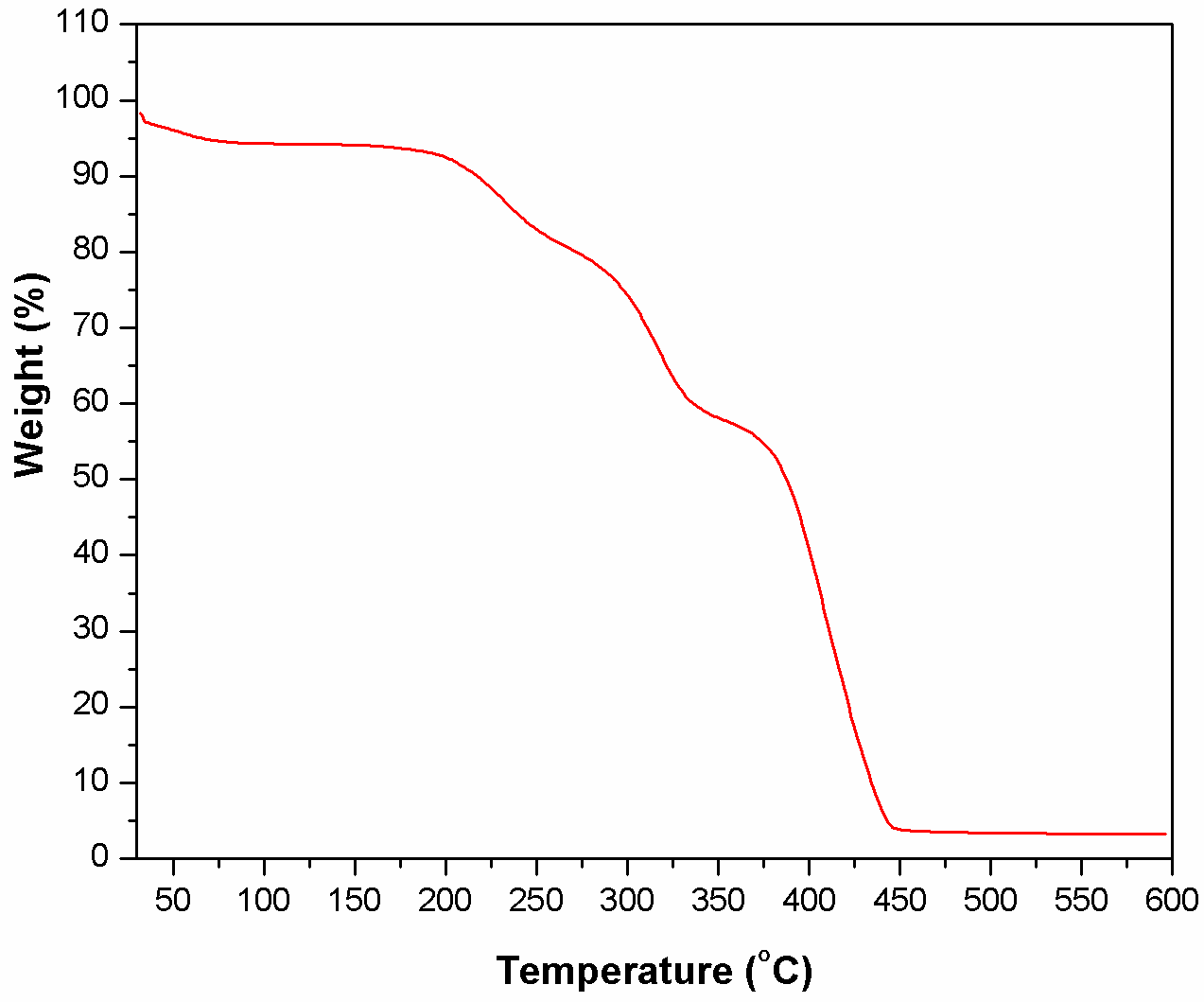


**Figure 2.** TGA data of CMMA3NF showing the heat stability of the 3D nanofibers.


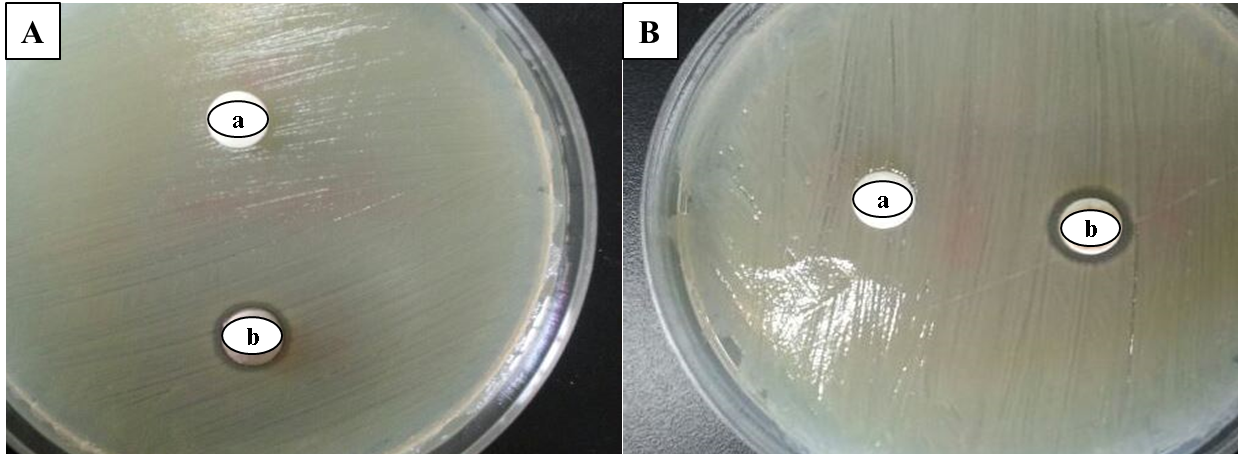


**Figure 3.** Antibacterial effect of CMMA 3NFs (b) compared to 2D NFs (a) on (A) staphylococcus aureus and (B) E-coli

The CMMA 3NFs showed an antibacterial effect to both Gram positive and Gram negative bacterial strains. S. aureus showed around a mean diameter of 14mm of inhibition diameter and E-coli showed around 17mm of inhibition diameter. . Meanwhile the 2D NFs didn’t show any antibacterial activity in both cases.


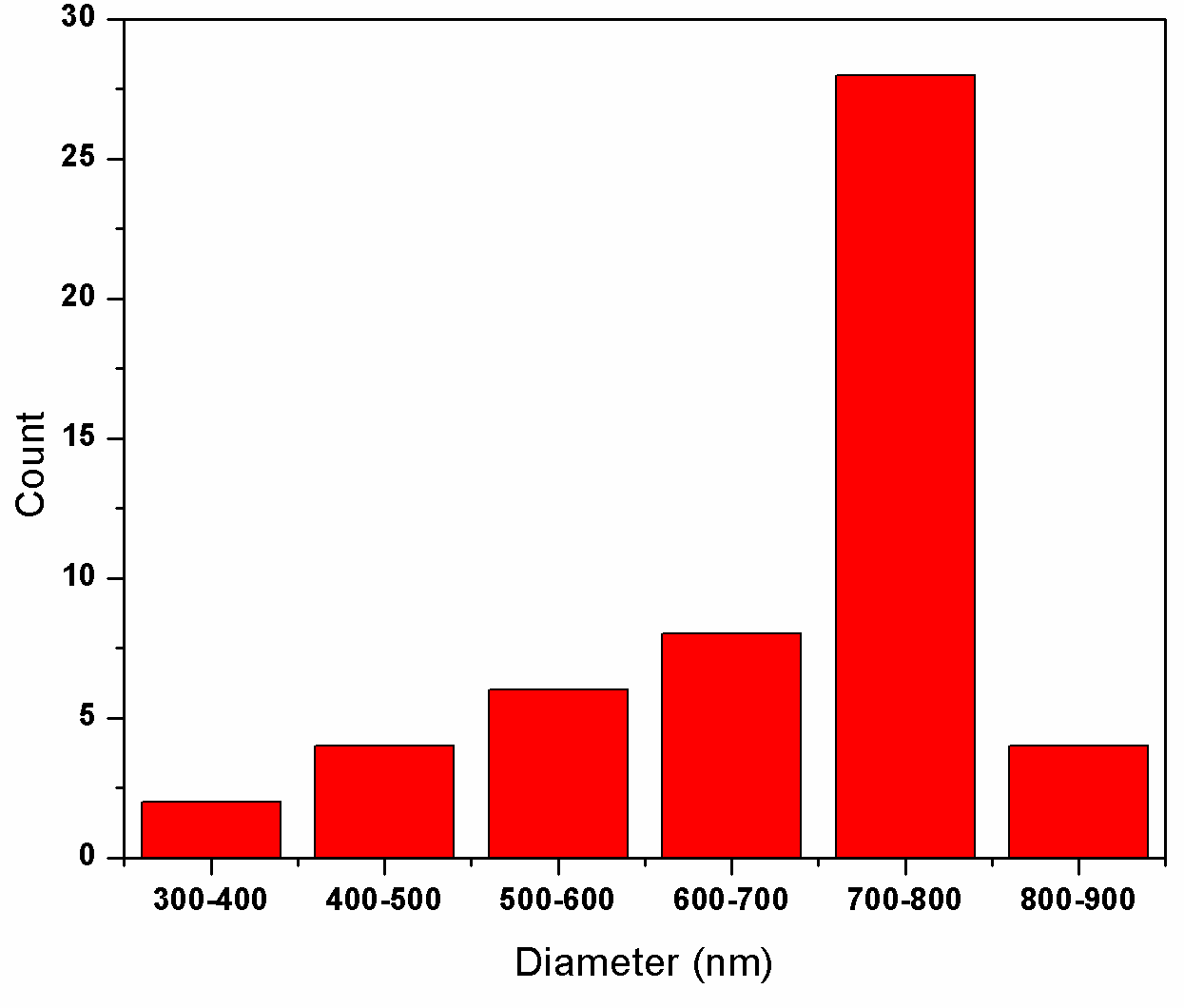


**Figure 4.** Fiber diameter distribution analysis showing the uniform fiber distribution at 700-800nm range


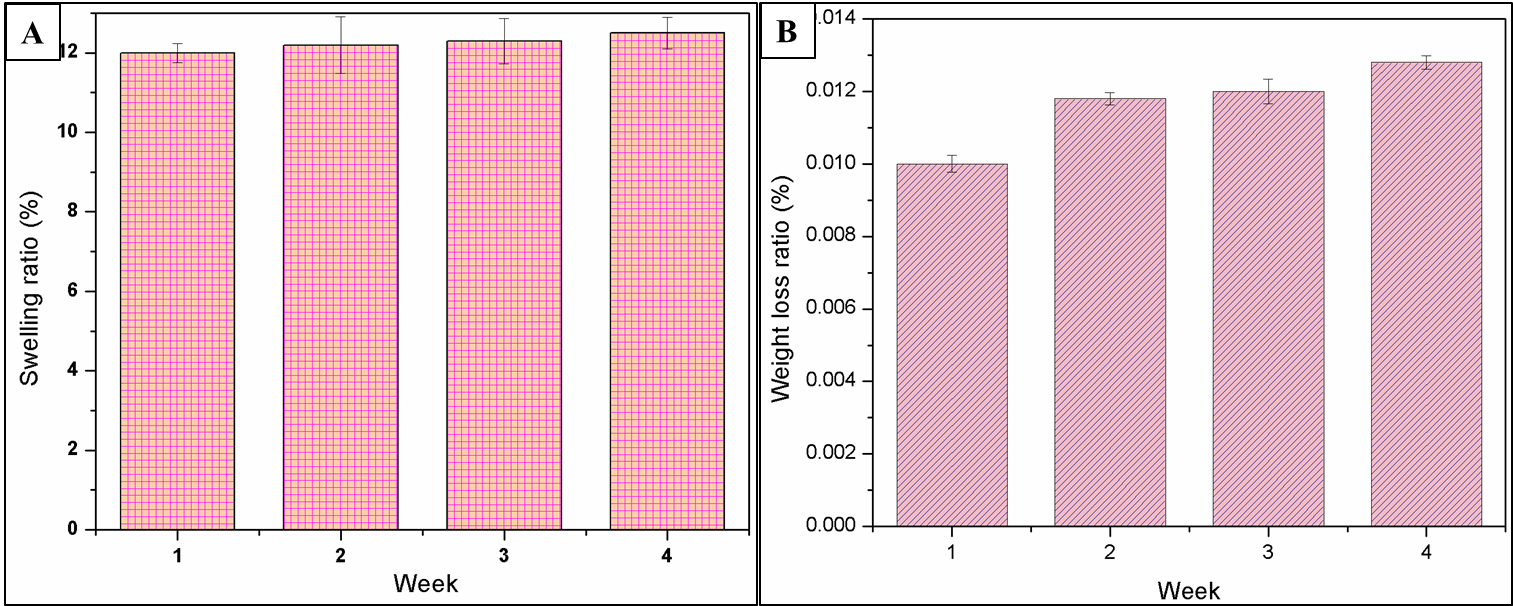


**Figure 5. (A)** Degradation property analysis and (B) the swelling ratio study of CMMA 3NF scaffolds in PBS
